# Chain-Based Communication in Cylindrical Underwater Wireless Sensor Networks

**DOI:** 10.3390/s150203625

**Published:** 2015-02-04

**Authors:** Nadeem Javaid, Mohsin Raza Jafri, Zahoor Ali Khan, Nabil Alrajeh, Muhammad Imran, Athanasios Vasilakos

**Affiliations:** 1 COMSATS Institute of Information Technology, Islamabad 44000, Pakistan; E-Mail: muhammadmohsinrazajafri@yahoo.com; 2 Internetworking Program, FE, Dalhousie University, Halifax B3J 4R2, Canada; E-Mail: zahoor.khan@dal.ca; 3 CIS, Higher Colleges of Technology, Fujairah Campus 4114, UAE; 4 B.M.T, C.A.M.S, King Saud University, Riyadh 11633, Saudi Arabia; 5 Deanship of E-Transactions and Communication, King Saud University, Riyadh 11692, Saudi Arabia; E-Mail: cimran@ksu.edu.sa; 6 Department of Computer Science, Kuwait University, Kuwait City 13060, Kuwait; E-Mail: vasilako@ath.forthnet.gr

**Keywords:** chain-based routing, cylindrical networks, routing protocols, UWSNs

## Abstract

Appropriate network design is very significant for Underwater Wireless Sensor Networks (UWSNs). Application-oriented UWSNs are planned to achieve certain objectives. Therefore, there is always a demand for efficient data routing schemes, which can fulfill certain requirements of application-oriented UWSNs. These networks can be of any shape, *i.e.*, rectangular, cylindrical or square. In this paper, we propose chain-based routing schemes for application-oriented cylindrical networks and also formulate mathematical models to find a global optimum path for data transmission. In the first scheme, we devise four interconnected chains of sensor nodes to perform data communication. In the second scheme, we propose routing scheme in which two chains of sensor nodes are interconnected, whereas in third scheme single-chain based routing is done in cylindrical networks. After finding local optimum paths in separate chains, we find global optimum paths through their interconnection. Moreover, we develop a computational model for the analysis of end-to-end delay. We compare the performance of the above three proposed schemes with that of Power Efficient Gathering System in Sensor Information Systems (PEGASIS) and Congestion adjusted PEGASIS (C-PEGASIS). Simulation results show that our proposed 4-chain based scheme performs better than the other selected schemes in terms of network lifetime, end-to-end delay, path loss, transmission loss, and packet sending rate.

## 1. Introduction

Seas and oceans have been used as a channel of communication, transportation and navigation from the very beginning. With the advancement in technology, different types of paradigms have been developed to enable the applications in water, such as ocean sampling, pollution monitoring and assisted navigation. One of these architectures is Underwater Wireless Sensor Network (UWSN). In this network, nodes are deployed underwater to perform specific tasks such as sensing of physical attributes of water. After sensing, sensor nodes transmit data to sinks/on-surface stations. They can also forward their data through intermediate nodes or underwater vehicles. One of the major subclasses of UWSN is Underwater Acoustic Sensor Network (UASN) in which acoustic signal is used for the communication between sensor nodes and the sinks. Acoustic signals can travel to longer distance due to lower frequency than radio waves. Such signals work well in water environment, however, acoustic signal causes high delay in data communication due to its speed of 1500 m/s. Acoustic signal has a frequency range between 10 kHz and 1 MHz.

UWSNs have huge number of applications such as seismic monitoring, submarine detection and oil spillage monitoring which require specific deployment strategies of sensor nodes. UWSNs may be of any shape, *i.e.*, rectangular, cylindrical or square. In this research work, we propose routing schemes specifically for application-oriented cylindrical networks. These applications require energy-efficient and delay-sensitive routing designs.

I.F. Akyildiz *et al.* [1] examine important challenges in acoustic communication and routing. They also address the routing challenges, according to the network protocol stack. They explore open research challenges in both 2-dimensional and 3-dimensional UWSNs. There are mainly two types of routing protocols in UWSNs *i.e.*, localization-based and localization-free routing protocols. In the first type, nodes perform data routing by using their location information. There are different ways to achieve localization information of the nodes, e.g., through GPS. In localization-free routing protocols, node does not have any localization information. S. Wang *et al.* [2] recommend an efficient way to achieve localization information of sensor nodes and environment mapping scheme utilizing robotic fish. It is mainly based on cooperative location and particle filter. Machine learning-based adaptive routing protocol for UWSNs, QELAR [3], discusses an outline for distributing residual energy equally among the nodes in order to compute reward function. In this scheme, reward function plays main role in selecting the optimal forwarder for sensor nodes. QELAR achieves enhanced network lifetime by using the reinforcement learning method. Another data-efficient scheme, Path Unaware Layered Routing Protocol for UWSNs, PULRP [4], provides a detailed algorithm to acquire low packet drop ratio along with decreased overhead of the network. In this scheme, nodes do not require localization information of their neighbor nodes. As mentioned earlier that acoustic signal experiences high delay in data communication due to its speed of 1500 m/s. Thereby, authors in [5] study queuing system with the assumption of slotted operation such that servers can serve in the beginning of the slot only. However, this assumption seems to be restrictive in many practical scenarios. Similarly, authors in [6,7] state that the back-pressure algorithm requires maintenance of separate queue for each destination, which prevents its implementation in large-scale networks.

**Figure 1.**
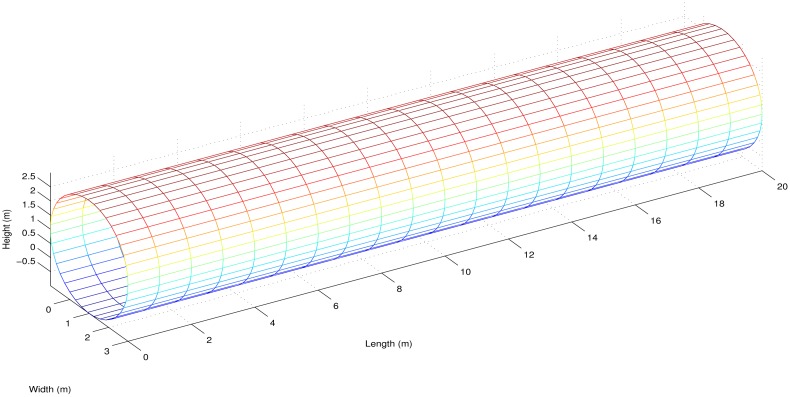
Cylindrical network.

In this paper, we propose 4-chain, 2-chain and single-chain based routing schemes for a cylindrical network as shown in Figure 1. The 4-chain routing scheme is introduced and the global optimum solution is found in terms of transmission distance. In the 2-chain routing scheme, we divide the deployed nodes in two separate regions and create 2-chain network to share the forwarding load. In the single-chain based routing scheme, there is a single multi-edge chain to transmit data to sink. The main objective behind this research is to improve network performance in terms of lifetime and throughput.

The rest of the paper is organized as follows: related work and motivation is discussed in Section 2. Section 3 gives the attenuation models to analyze the energy consumption, end-to-end delay and transmission loss of network. Sections 4–6 contain a brief description of 4-chain, 2-chain and single-chain based routing schemes, respectively. Simulation results are presented in Section 7. Finally, the paper is concluded in Section 8.

## 2. Related Work and Motivation

In recent years, a lot of research has been carried out on routing protocols of UWSNs. S. Tolba *et al.* [8] suggest an energy-efficient routing protocol which jointly utilizes single-hop and multi-hop communication; however, it also allows high delay in data transfer. H. Luo *et al.* [9] suggest another application-oriented routing protocol for UASNs. They propose an energy-harvesting technique specifically for the underwater moored system. They also formulate the model for energy consumption and path loss in underwater environemnt. Hop-by-Hop Vector-Based Forwarding for underwater sensor networks (HHVBF) [10] is another localization based protocol which assumes vectors formation between the transmitting and receiving nodes. In this way, it reduces control overhead and end-to-end delay of the network. In [11], authors analyze the path loss and the effects of wave movement on acoustic signal. They also provide improved computational model for path loss of a signal.

Collection of data through chain formation is an efficient way in terrestrial Wireless Sensor Networks (WSNs). It saves the energy of sensor nodes and minimizes delay. In rectangular and cylindrical network, chain formation offers improved performance by finding both local optimum and global optimum solutions for data gathering. There are many routing protocols in WSNs which work on the principle of chain formation. PEGASIS [12] is one of the chain based protocols. In this scheme, greedy algorithm is implemented. Initially, chain leader node if found out which is the farthest node from BS. Each node then communicates with its closet neighbor. Soon after chain establishment, a node with highest residual energy is selected as chain leader node. The chain leader node is responsible to directly communicate with BS as it gathers data from all other chain member nodes. Thus, the energy consumption cost is minimized to some extent. However, the length of over all routing path is somewhat increased which leads to increased energy consumption. There are many improved versions of PEGASIS. In [13], authors propose an improved multi-edge chain to minimize distant neighbor problem in which a long link is created in the chain. Similarly, [14] considers distance, energy and congestion while constructing chain. As a result, these schemes improve the network lifetime in an efficient manner.

Multi-Path Transmission (MPT) proposed in [15], minimizes the challenges at the physical layer by proposing source-initiated and power-controlled transmission. It also provides reactive routing for on-demand data applications. Another reactive routing protocol, improved Adaptive Mobility of Courier nodes in Threshold-optimized Depth-based-routing (iAMCTD) [16] improves the network throughput and largely minimizes packet drop ratio by using its formulated Forwarding Functions *FFs*. To reduce network lifetime, mobile courier nodes are utilized in UWSNs. In [17], authors use autonomous underwater vehicles to minimize end-to-end delay and is specifically designed for delay-sensitive applications for UWSNs. In UWSNs, sensor nodes also transfer the data towards the underwater vehicles. They use both direct transmission and multi-hop transmission. In another application, nodes sense the moving targets through UASNs. X. Wang *et al.* [18] suggest a scheme to track moving targets on the basis of particle filter technique. They also combine filter technique and interacting-model method.

Moreover, there are some energy-efficient data routing schemes such as Link-State Based routing (LSB) [19] and Round-Based Clustering (RBC) [20]. These protocols minimize the major problems in acoustic routing such as energy dissipation of nodes, high end-to-end delay and high path loss by using different methods. Some of these techniques, tackle the problems more realistically than the other schemes by considering the node's mobility and shallow water conditions. In UWSNs, there is a major problem of high transmission collisions, which can not be handled by routing protocols. In [21], authors decrease the transmission collisions by suggesting the multichannel Medium Access Control (MAC) protocol. Depth-Based routing in underwater sensor networks (DBR) [22] is a trivial routing scheme in which sensor nodes use their depth information to transfer data towards the on-surface station. Nodes do not require information of their location, however, they identify their depth by using depth sensor.

In acoustic communication, signal quality is largely affected by lower bandwidth efficiency and frequency. Therefore, in order to achieve increased throughput, frequency scaling is used. In [23], authors analyze the effects of frequency scaling on channel capacity. They also utilize multi-hop communication in dense UASNs to achieve high quality signal. Another way to increase the network lifetime of UASNs is by employing Remotely Powered UASN (RPUASN). In [24], authors suggest paradigm of RPUASN in which sensor nodes harvest along with storing the power supplied by an external acoustic source. In [25], authors employ both the ant colony optimization algorithm and artificial fish swarm algorithm to achieve a global solution for efficient data gathering. It also decreases delay and energy consumption of nodes in UWSNs.

There is large number of routing protocols designed for application-oriented UWSNs. Applications such as oil spillage monitoring and seismic monitoring require cylindrical deployment of sensor nodes. However, there is a lack of energy efficient routing schemes for the cylindrical networks in UWSNs. In terrestrial WSNs, single-chain based routing schemes such as PEGASIS does not perform well in the underwater environment. Challenges such as increased energy consumption and unbalanced load distribution may only be tackled by improving chain-based routing schemes. There is also a problem of long link in the existing chain-based schemes such as PEGASIS.

## 3. Acoustic Models

In UWSNs, an acoustic signal is used for the communication between the sensor nodes, which tackle the challenges of the aqueous environment in a better way. In this section, acoustic models are presented to calculate energy consumption, delay, transmission loss and other important performance parameters in the acoustic environment.

### 3.1. Energy Consumption Model

To analyze the energy consumption model [9] for acoustic communication, we first use the passive sonar equation to calculate Signal-to-Noise Ratio (SNR) in an acoustic channel.

(1) SNR=SL−TL−NL+DI≥DT

In the above equation, *SL* and *TL* denote Source Level and Transmission Loss, respectively. Moreover, *NL* is Noise Loss, *DI* is Directive Index and *DT* is the Detection Threshold of the sonar. Units of all the quantities are in *dBreµPa*.

Transmission loss may be computed by using Thorp model [26] as follows:

(2) $$TL=10\log(d)+\alpha d\times 10^{-3}$$

where, *α* is the absorption coefficient and *d* is the distance between sender and receiver nodes.

Another important factor, NL [27], consists of four noise components, which are calculated by using the equations given below. *NL* depends upon frequency of the signal:

(3) $$10\log(N_{t}(f))=17-30\log(f)$$

(4) $$10\log(N_{s}(f))=40+20(s-0.5)+26\log(f)-60\log(f+0.03)$$

where *s* is a shipping constant and *w* is a wind constant.

(5) $$10\log(N_{w}(f))=50+7.5w^{1/2}+20\log(f)-40\log(f+0.4)$$

(6) $$10\log(N_{th}(f))=-15+20\log(f)$$

where, *N_w_*(*f*), *N_s_*(*f*), *N_t_*(*f*) and *N_th_*(*f*) show the noise produced due to wind, shipping, turbulence and thermal activities, respectively. All these factors largely depend on frequency (*f*) as noise increases with the increase in frequency of signal. *NL* is calculated as:

(7) $$NL=N_{t}(f)+N_{s}(f)+N_{w}(f)+N_{th}(f)$$

*SL* can also be calculated by using passive sonar equation.

(8) SL=SNR+TL+NL−DI

Transmitted signal Intensity, *I_T_*, may also be calculated by using SL.

(9) $$I_{T}=10^{SL/10}\times 0.67\times 10^{-18}$$

Source transmitted power, *P_T_*(*d*), can be calculated by using the following equation:

(10) $$P_{T}(d)=2\pi \times 1m\times H\times I_{T}$$

In the above equation, *H* shows the depth of the network. *P_T_*(*d*) can also be written as:

(11) $$P_{T}(d)=2\pi H\times 1m\times H\times 10^{SL/10}\times 0.67\times 10^{-18}$$

Energy dissipation, *E_TX_*(*k*, *d*), in sending *k* bits over a distance *d* is given as follows:

(12) $$E_{TX}(k,d)=P_{T}(d)\times T_{TX}$$

where *T_TX_* is the transmission time in seconds.

### 3.2. Delay Computation Model

**Figure 2.**
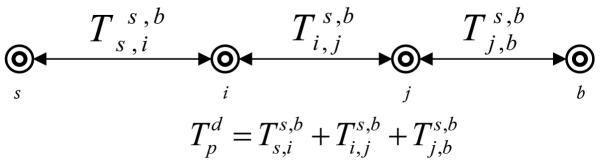
Propagation delay in multi-hop communication.

In this section, we suggest an analytical model to calculate end-to-end delay in data transmissions. We study the effects of acoustic channel characteristics on the speed and propagation delay of the signal. The propagation delay of acoustic signal is five times greater than the RF signal. Figure 2 shows the description of propagation delay.

The end-to-end delay between the sender and receiver is given by:

(13) $$T_{E-E}=(n+1)(T_{tx})+n(T_{rx})+T_{p}^{d}$$

where *T_tx_* and *T_rx_* are the consumed transmission and reception time of a packet. *n* is the number of hops for a specific packet, whereas, $T_{p}^{d}$ is the overall propagation delay of packet from source to sink and is expressed as:

(14) $$\begin{array}{cc} T_{p}^{d}=T_{s,i}^{s,b}+\underset{i,j\in n}{\text{∑}}T_{i,j}^{s,b}+T_{j,b}^{s,b}, & n\geq 2\wedge i,j\in n \end{array}$$

(15) $$\begin{array}{cc} T_{p}^{d}=T_{s,i}^{s,b}+T_{i,b}^{s,b}, & n=1\wedge i\in n \end{array}$$

(16) $$\begin{array}{cc} T_{p}^{d}=T_{s,b}^{s,b}, & n=0 \end{array}$$

Equations (14), (15), and (16) show the computations of propagation delay for multi-hop, single-hop and direct communication, respectively.

Propagation delay between two communicating nodes has been calculated in [28] and is given as:

(17) $$T_{p}=d/q$$

where, *d* is the distance between the sender and receiver in *m* and *q* is the speed of signal which is calculated as follows [11]:

(18) $$q=1449.05+45.7t-5.21t^{2}+0.23t^{3}+\left(1.333-0.126t+0.009t^{2}\right)\left(S-35\right)+16.3z+0.18z^{2}$$

(19) t=T/10

In the above equations, *T* is the temperature in °*C, S* is salinity and *z* is the depth in *km*.

### 3.3. Acoustic Propagation Models

Acoustic propagation models examine path loss and transmission loss in an aqueous channel. Recently proposed models such as MMPE [27] and the thorp model consider depth, signal height and combined noises in aqueous environment. However, some models regard frequency and bandwidth efficiency as the deciding factors for variations in path loss and delay. In the following subsections, we analyze two main acoustic propagation models that compute the combined losses in UWSN.

#### 3.3.1. Thorp Formula

Thorp formula considers the acoustic signal propagation as a molecular movement of signal towards its adjacent particles. It predicts the amount of gradual decrease in signal intensity, as the signal propagates towards the destination node. However, its main emphasis is on the bandwidth efficiency. It proposes an absorption coefficient (*α*) which is a function of frequency and distance of transmitted acoustic signal. It also suggests a model for the calculation of combined acoustic absorption loss. Thus, at a given frequency *f*, thorp model calculates the total absorption loss as follows:

(20) $$10\log\alpha (f)=\{\begin{array}{ll} 0.11f^{2}/(1+f^{2})+44f^{2}/(4100+f) & \\ +2.75*104f^{2}+0.003, & f\geq 0.4\\ 0.002+0.11(f/(1+f))+0.011f, & f<0.4 \end{array}$$

where *α*(*f*) is measured in dB/km and *f* in kHz. By using above calculated loss, we calculate the value of *α* as follows:

(21) $$\alpha =10^{\alpha (f)}/10$$

All the tunable parameters are given in *dBreμPa*. The total attenuation can be calculated by adding absorption and spreading loss:

(22) 10logA(l,f)=k∗10log(l)+l∗10log(α(f)),

The first term in Equation (22) shows the spreading loss and the second term is the absorption loss. *l* is the transmission distance in meters. The spreading coefficient *k* shows the geometry of the signal propagation. (*i.e.*, *k* = 1 is cylindrical, *k* = 2 is spherical, and *k* = 1.5 is practical spreading [29]).

#### 3.3.2. Monterey-Miami Parabolic Equation

Monterey-Miami Parabolic Equation (MMPE) is also an accurate model that computes channel losses in acoustic channel. It is formulated by using the main principle of wave equation. It is highly complex model, however more accurate than the Thorp model. It also shows the impacts of variations in the depth information of the sender and receiver nodes on the signal quality. Moreover, it considers the Euclidean distance between the communicating nodes and the frequency of the transmitted signal.

The basic formula of MMPE model is given below [30]:

(23) $$PL(t)=m(f,s,d_{A},d_{B})+w(t)+e()$$

where:

*PL*(*t*): propagation loss occurs during transmission from node A to node B.
*m*(): propagation loss with random and periodic components; obtained from regression of MMPE data.
*f*: frequency of transmitted acoustic signal in kHz.
*s*: Euclidean distance between node A and node B in meters.
*w*(*t*): periodic function to approximate signal loss due to wave movement.
*d_A_*: sender's depth in meters.
*d_B_*: receiver's depth in meters.
e(): signal loss due to random noise error.

The first term of the Equation (23) calculates the propagation loss caused by the random and periodic components. It also performs nonlinear regression on the data to obtain *A*(*n*) coefficients. By using the resulting data, *m*(*f*, *s*, *d_A_*, *d_B_*) function computes the propagation loss [31] as follows:

(24) $$m\left(f,s,d_{A},d_{B}\right)=\log\left(\left|\frac{{\left(\frac{s}{0.9}\right)}^{A0}d_{A}^{A9}s^{A7}{\left({\left(d_{A}-d_{B}\right)}^{2}\right)}^{A10}}{{\left(s*d_{B}\right)}^{10A5}}\right|\right)+\left(f^{2}\left(\frac{A1}{1+f^{2}}+\frac{41}{4100+f^{2}}+0.002\right)+0.003\right)*\left(\frac{s}{914}\right)+A6*d_{B}+A8*s$$

In the second part of the Equation (23), MMPE model estimates the losses caused due to wave motion. For this purpose, it considers the sinusoidal movement of the water particles around the acoustic signal. The *w(t)* [30] function considers scale factor function, wavelength and wave effects function to predict the signal loss caused by the wave movement. It can be mathematically written as:

(25) $$w\left(t\right)=h\left(l_{w},t,d_{B},h_{w},T_{w}\right)*E\left(t,T_{w}\right)$$

where:

*h(s)*: scale factor function.
*l_w_*: ocean wavelength in meters.
*h_w_*: wave height in meters.
*d_B_*: receiver's depth in meters.
*T_w_*: wave period in seconds.
*E*(): function of wave effects in nodes.

As, we assume the continuous node's movement in aqueous environment, so *h(s)* depicts the effect on wavelength of transmitted signal due to receiver node's movement. It also scales the movement of signal with the distance. We compute scale factor by using the following formula:

(26) $$h\left(T_{w},l_{w},t,h_{w},d_{B}\right)=\left(\frac{\left(h_{w}\left(1-\left(\frac{2d_{B}}{l_{w}}\right)\right)\right)}{0.5}\right)*\left|\sin\left(\frac{2\pi \left(\mathrm{mod}\left(T_{w}\right)\right)}{T_{w}}\right)\right|$$

Third term of Equation (23), *e*( ) describes the random background noise. In order to estimate the noise in dense conditions, the random noise function used by *e*( ) follows a Gaussian distribution. The random noise function is based on the proportion of the distance between communicating nodes and the source transmitter range.

(27) $$e\left(\right)=20\left(\frac{s}{s_{\max}}\right)R_{N}$$

where:

*e*(): random noise function.
*s_max_:* maximum transmission range in meters.
*R_N_:* random number from a Gaussian distribution centered at 0 with variance 1.

## 4. 4-Chain Based Routing

Subject to the improvement in the energy efficiency of UWSNs, we propose the 4-Chain based routing protocol. The proposed protocol works on the basic principle of divide-and-conquer. We first divide the whole network into cylinders and the divide the entire cylindrical network area into sub-regions. In each sub-region, the algorithm is independently implemented such that the overall methodology uses shorter parallel routes that is converse to the lengthy rout of PEGASIS and C-PEGASIS. Thus, the energy consumption is reduced leading to improvement in terms of network lifetime, which is, of course, one of the most wanted parameters of tiny battery operated networks. Data routing is performed in a cylindrical network by using four interconnected chains of sensor nodes. Firstly, we create chains and then interconnect them to find global optimum paths instead of the local optimum paths for data routing. Detailed description of the proposed protocols is in the following subsections.

### 4.1. Network Model and Assumptions

**Figure 3.**
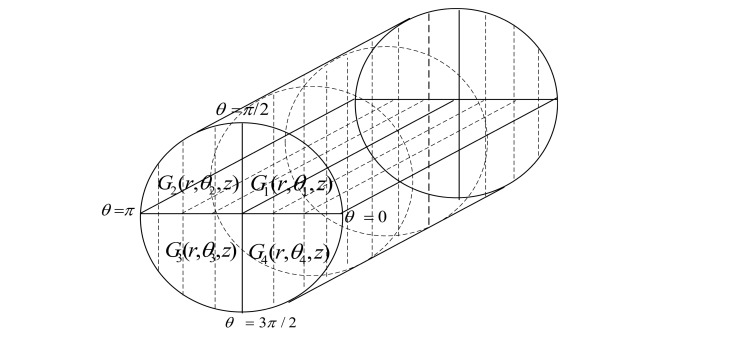
Regions formation in 4-chains based scheme.

We assume a cylindrical network for an acoustic environment in order to design application-oriented network. We divide the network into four regions and also create four groups of sensor nodes on the basis of these regions. Figure 3 shows the formation of regions in the assumed network on the basis of ranges of *θ*.

In our scheme, we assume ℵ number of nodes with the same amount of initial energy which are randomly deployed in the region of 1000 *m*^2^. The transmission range of each sensor node is *R*. Four ranges of *θ* defining the basis for regions are as follows:

$$\begin{array}{rr} \theta_{1}\in [0,\pi /2), & \theta_{2}\in [\pi /2,\pi )\\ \theta_{3}\in [\pi ,3\pi /2), & \theta_{4}\in [3\pi /2,0]\\ G_{1}(r,\theta_{1},z), & G_{2}(r,\theta_{2},z),\\ G_{3}(r,\theta_{3},z), & G_{4}(r,\theta_{4},z), \end{array}$$

Here, *G1, G2, G3* and *G4* show the groups of nodes, randomly located in the four regions, and their ranges in terms of *θ*. Nodes in the all four groups have the same ranges for *r* and *z*. Creation of chains starts in all the regions through token passing approach. Four separate chains are created as the token passes from the farthest node from sink to the nearest node to the sink. We compute the lengths of four chains as follows:

(28) $$l_{i,i+1}^{j}=\left(n_{i+1}^{j}-n_{i}^{j}\right)$$

In the above equation, $n_{i}^{j}$ denotes the *ith* node present in the *jth* chain of the network, whereas, $l_{i,i+1}^{j}$ is the length of chain between any two consecutive nodes in *jth* chain.

(29) $$L_{G}^{j}=\overset{N^{j}-1}{\underset{i=1}{\text{∑}}}\left(n_{i+1}^{j}-n_{i}^{j}\right)$$

In Equation (29), $L_{G}^{j}$ shows the total length of the *jth* chain which consists of *N^j^* nodes.

### 4.2. Protocol Operations

For the sake of simplicity, we divide the protocol operation into four phases. Since the newly deployed nodes do not have a reliable communication infrastructure, the information sharing needs to be initiated as soon as the nodes are deployed. In this regard, we introduce the initialization phase prior to the protocol operation. In this phase, nodes (with the basic aim to update all the network configuration) broadcast their location information towards the sink. Thus, the chances of conducting successful communications increase. In the second phase, sensor nodes identify their regions on the basis of *θ* to form chains. Then the nodes interconnect with the chains by finding their nearest neighbors in the other chains. Finally, the data is transferred towards the sink through global optimum paths, which definitely increases the throughput of overall network, reduces the end-to-end delay, and saves the energy consumption prolonging the network life time.

#### 4.2.1. Formation of Chains

Once the network is converged, and nodes become fully aware of coordinates of all nodes in the network, the protocol starts its first phase. In the first phase, chain creation starts with the farthest node from the sink. Every node identifies its nearest node and connects with it. Sink transmits a token towards the farthest node of the chain. The chain is created as the token is passed towards sink through the intermediate nodes. In this way, four chains are created as the nodes identify their regions and continue to insert other nodes in their respective chains by using shortest path selection.

We also formulate the problem of chain creation by using mixed integer linear programming. In this model, our objective function is to minimize the total transmission distance, *D*, of all the chains in a round. A round is a time in which all the nodes transmit their data towards the sink. Total energy consumption of all the nodes in a single round is directly proportional to *D*.

(30) $$\begin{array}{cc} \mathrm{Minimize} & D \end{array}$$

subject to

(31) $$\begin{array}{cc} \text{Constraint}\;1. & D\geq \overset{4}{\underset{j=1}{\text{∑}}}D^{j} \end{array}$$

(32) $$\begin{array}{cc} \text{Constraint}\;2. & D^{j}\geq \overset{N^{j}-1}{\underset{i=1}{\text{∑}}}\left(n_{i+1}^{j}-n_{i}^{j}\right) \end{array}$$

(33) $$\begin{array}{cc} \text{Constraint}\;3. & D\geq \overset{4}{\underset{j=1}{\text{∑}}}\overset{N^{j}-1}{\underset{i=1}{\text{∑}}}\left(n_{i+1}^{j}-n_{i}^{j}\right) \end{array}$$

(34) $$\begin{array}{cc} \text{Constraint}\;4. & d_{i}^{j}= \end{array}(n_{i+1}^{j}-n_{i}^{j})$$

The objective function, *D* could be achieved fulfilling the following constraints in the above equations:

- Constraint 1 shows that the total transmission distance; *D* is greater than or equal to the sum of distances *D^j^* of all the interconnected chains,
- Constraint 2 shows that the total transmission distance of a *jth* chain is larger than or equal to the sum of the distances between the nodes of the chain, and
- Constraint 3 explains the total transmission distance in a detailed way. The last constraint defines the transmission distance between any parent and child nodes in the chain.

#### 4.2.2. Election of Chain Heads

In this phase, the chain head is selected on the basis of *W*. All the nodes compute this factor by using the Equation (35). In this mechanism, the network compares the value of *W* of all the nodes in the chain. The node with the highest *W* factor in the chain is selected as a primary chain head. Each node *i* calculates its distance with the parent node and then compares it with the distance to the sink. If the later distance is shorter, the node *i* acts as a secondary chain head and sends the collected data to the sink, instead of transferring it to the parent node.

(35) $$W_{i}=E_{i}/S_{i,s}$$

where, *E_i_* is the residual energy of *ith* node and *S_i,s_* is the distance between node *i* and sink.

#### 4.2.3. Formation of Interconnection between Chains

In order to find a global optimum path, every node *i* compares its distance from its parent node *p_i_* with the distance from the nearest neighbor in the other chains. If the neighbor in the other chain is closer than *p_i_*, the node *i* transmits data to neighbor instead of sending it to the *p_i_*. It also updates its parent node on the basis of this comparison. We also formulate this selection of interconnecting nodes between the chains as:

(36) $$N_{i}^{R}=\{k_{i}\in ℵ|d(i,k_{i})\leq R\}$$

In the Equation (36), $N_{i}^{R}$ is the set of neighbors of node *i*, present in its transmission range, *R*.

(37) $$N_{i}^{j}=\{k_{i}^{j}\in N_{i}^{R}\wedge k_{i}^{j}\in G_{j}\}$$

Here, $N_{i}^{j}$ is the set of neighbors of node *i*, which are present in its chain and $\bar{N_{i}^{j}}$ is the set of neighbors not present in the chain of node *i*. *G_j_* is the group of nodes present in *jth* chain.

(38) $${\bar{N}}_{i}^{j}=\left\{{\bar{k}}_{i}^{j}\in N_{i}^{R}\wedge {\bar{k}}_{i}^{j}\notin G_{j}\right\}$$

Any node *i* selects a parent node *p_i_* from the other chain, if the nearest node $k_{i}^{j}$ in its chain is farther than the node ${\bar{k}}_{i}^{j}$ in the other chain.

(39) $$p_{i}=\{\begin{array}{cc} k_{i}^{j} & if\;d_{i}^{j}<{\bar{d}}_{i}^{j}\\ {\bar{k}}_{i}^{j} & \text{otherwise} \end{array}$$

#### 4.2.4. Data Transmission

**Figure 4.**
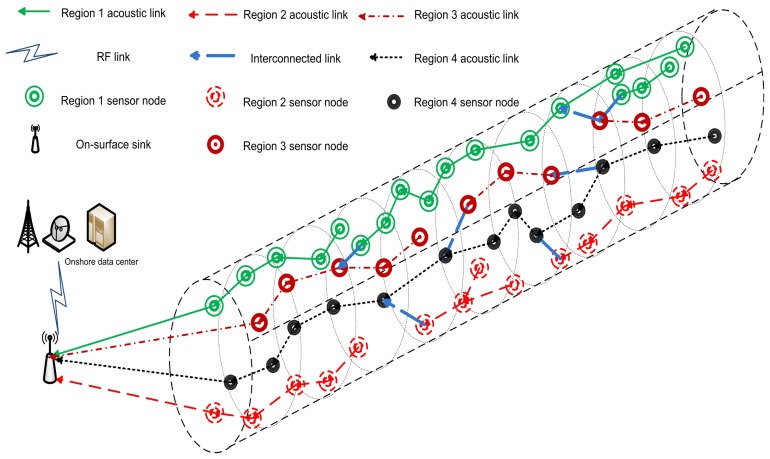
4-chain based routing.

When all the pre-requisites subject to chain(s) organization are performed, all of the nodes transmit their data towards their parent nodes in their allocated Time Division Multiple Access (TDMA) based schedules. To avoid the synchronization problem, we assume that all the TDMA based schedules are assigned by sink. The parent nodes aggregate the data and forward it towards the sink. Later on, data forwarding load is divided between the chain heads of four chains. Problems of distant forwarder node and *long link* are solved by the interconnection of chains. When all the data transmissions are conducted, next phase begins with the network configuration that plays a vital in deciding the next chain leader node, parent nodes, child nodes, *etc*. Figure 4 shows the data transmission for a 4-chain based routing scheme.

## 5. 2-Chain Based Routing

In this scheme, we perform data routing in a cylindrical network by using two interconnected chains of sensor nodes. We create two chains and interconnect them to find a global optimum path instead of the local optimum neighbors for data routing. We divide the network into two regions on the basis of *θ*. The nodes present in these regions form two separate chains.

### 5.1. Network Model and Assumptions

**Figure 5.**
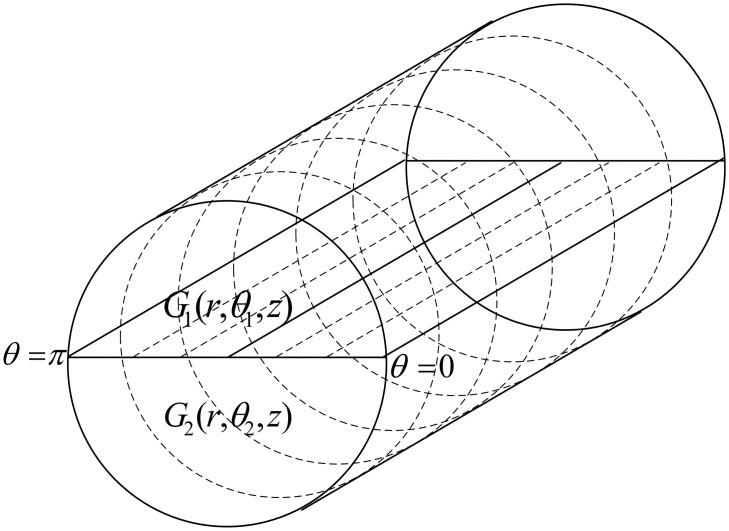
Regions formation in 2-chain based scheme.

**Figure 6.**
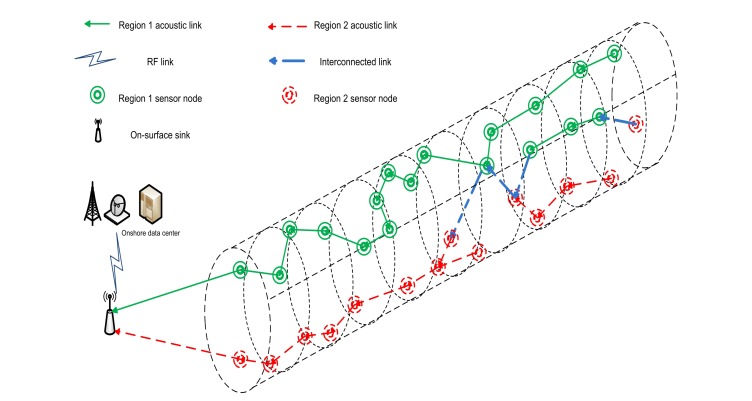
2-chain based routing.

In the 2-chain model, we divide the network in two separate regions as shown in Figure 5. Then we create two groups of sensor nodes on the basis of these regions. We suppose ℵ number of nodes randomly deployed in the region of 10,000 *m*^2^. Two ranges of *θ* define the regions and can be written as follows:

$$\begin{array}{rr} \theta_{1}\in \left[0,\pi \right), & \theta_{2}\in \left[\pi ,0\right]\\ G_{1}\left(r,\theta_{1},z\right), & G_{2}\left(r,\theta_{2},z\right) \end{array}$$

where *G*_1_ and *G*_2_ show the groups of nodes randomly located in the two regions. Following the previous scheme, formation of chains starts using token passing approach after the formation of the regions. Figure 6 shows the data transmission in 2-chain based routing scheme.

### 5.2. Protocol Operations

We divide the operation of protocol in two main phases. In the first phase, nodes broadcast their location information towards the sink and their neighbors. Sensor nodes find their regions on the basis of *θ*. In the second phase, chains are created by selecting nearest nodes. Chains are then interconnected and finally the data is transmitted towards the sink through global optimum paths.

#### 5.2.1. Formation of Chains and Election of Chain Head

Chain creation starts from the farthest node from the sink. Every node finds its nearest node which is not already connected to chain. Sink transmits a token towards the farthest node of the chain. The farthest node transmits the token in the reverse direction towards the sink. Each node uses shortest path selection algorithm to find its nearest node in the chain formation. The primary chain head is selected on the basis of *W*. After the chain formation, every node identifies its child and parent node. The node receiving data from the *ith* node is parent of *i*, whereas, the node sending data to the *ith* node is its child node.

#### 5.2.2. Chains Interconnection and Data Transmission

In order to find a global optimum path, every node compares the distance from its parent node with the distance from its nearest neighbor in the other chains. If the respective nearest neighbor is closer than the parent node, then node transmits data to the respective neighbor node. It also updates its parent nodes on the basis of this comparison. We can say that any node *i* selects a parent node *p_i_* in the other chain, if the nearest node $k_{i}^{j}$ in its chain is farther than the node ${\bar{k}}_{i}^{j}$ in the other chain. In the last phase of the scheme, all the nodes transmit their data towards their parent nodes which forward it to the sink.

## 6. Single-Chain Based Routing

In this scheme, data routing is performed in a cylindrical network by using a single chain of sensor nodes. Every node finds its nearest neighbor and connects with it. In this way, local optimum neighbors of sensor nodes are identified. However, it does not achieve the global optimum data transmission as there exists a *long link* problem. During network initialization, nodes broadcast their location information towards the sink and their neighbors. In the first phase, chain creation starts from the farthest node from the sink by using token passing approach.

Every node finds its nearest node which is not already connected to the chain. In the token passing approach, the sink transmits a token towards the farthest node of the chain. The chain is formed as the token is passed towards sink through the intermediate nodes. The primary chain head is selected on the basis of *W*. All nodes compute the value of *W* by using their residual energy and distance from the sink. The sink compares the *W* of all the nodes in the chain. The node with the highest *W* is selected as a primary chain head. All the nodes transmit their data towards the sink through intermediate nodes.

**Figure 7.**
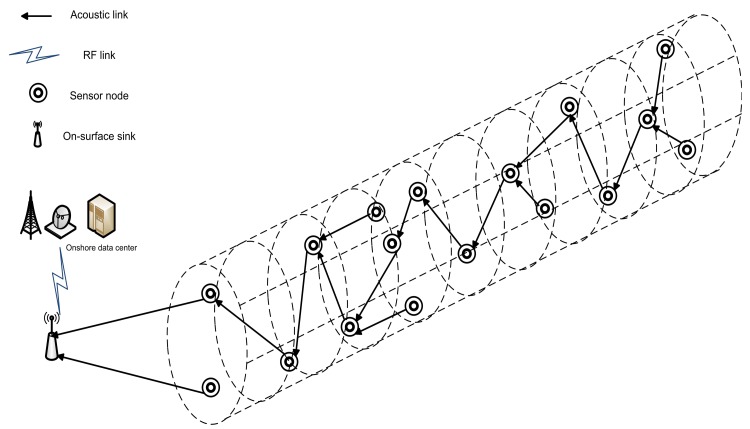
Single-chain based routing.

Then each node *i* compares its distance from the parent node and distance to the sink. If the later distance is less, the node *i* acts as a secondary chain head and sends the collected data to the sink, instead of transferring it to its parent node. In the last phase, nodes transmit their data towards their respective parent nodes. Data is forwarded to the sink through primary and secondary chain heads. Figure 7 shows the data transmission in single-chain based routing scheme.

## 7. Performance Evaluation and Analysis

In this section, we study the performance of 4-chain, 2-chain, single-chain, PEGASIS [12] and C-PEGASIS [14] routing schemes in realistic acoustic environment. Loss caused by shipping, thermal and turbulence noise is also computed. In simulations, we have assumed a cylindrical network of area 10,000 *m*^2^ with the sink stationed at one end of the network. There are 100 sensor nodes randomly deployed in the network. Each sensor node has a transmission range of 250 m. By following the convention of existing routing protocols of UWSNs, we use an acoustic modem of LinkQuest UWM1000 [32] having a bit rate of 10 kbps. Sensor nodes transmit a data packet after every 16 s. According to the specifications of the modem, the power consumption in transmission, reception, and idle mode is 2 W, 0.1 W, and 10 mW, respectively. The size of the data packet and control packet is 50 bytes and 8 bytes, respectively. The initial energy of the sensor node is set as 10 joules.

**Figure 8.**
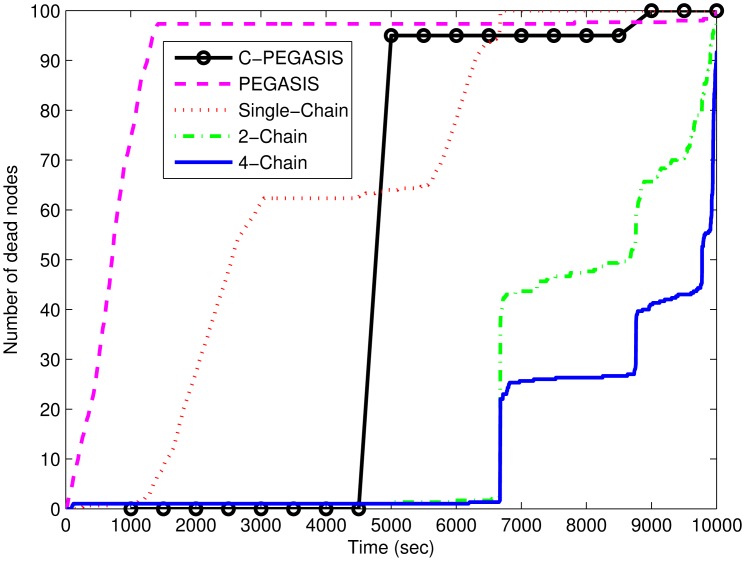
Number of dead nodes in 4-chain, 2-chain, single chain-based routing and PEGASIS.

Figure 8 shows that the network lifetime of the single-chain based scheme is much smaller than that of the other two schemes. In single-chain routing, there is large forwarding burden on the single chain head; however, in the other two schemes, the load is divided among two or four chain heads. Although residual energy is also among the factor which is used in the selection of chain head, it does not improve the network lifetime in single-chain as in the 2-chain and 4-chain based routing schemes. In 4-chain routing, network lifetime is higher than 2-chain based routing scheme. This is because of more chain heads in 4-chain based scheme than in 2-chain based scheme. The problems of the presence of distant neighbor and *long link* are very important in chain-based routing. In the multi-chain based routing, these problems are removed by using the interconnection between the chains. Nodes having distant neighbors are connected to their nearest neighbor in any other chain. In PEGASIS, quick death of nodes in the early time duration causes a shorter stability period. The chain head having larger distance from the sink than the other nodes consumes a large amount of energy in data aggregation and transmission. Moreover, there is a trade-off between the stability period and network lifetime. Therefore, PEGASIS has a longer lifetime than single-chain based routing; however, it has smaller stability period than the later scheme. The nodes nearer to the sink remain alive for a longer duration than the other nodes. Congestion consideration in C-PEGASIS prolongs its stability period and network lifetime to some extent (*i.e.*, better than PEGASIS and single chain); however, this impact is less than that of the 2-chain based and the 4-chain based schemes.

**Figure 9.**
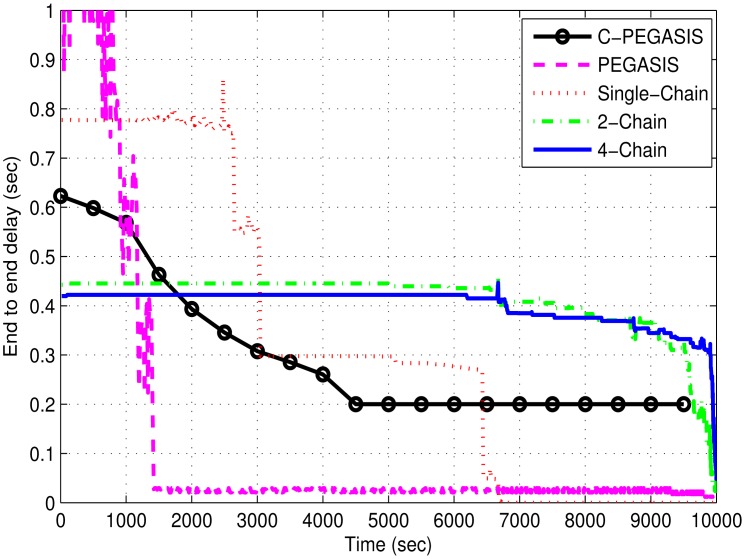
End-to-end delay (sec) in 4-chain, 2-chain, single chain-based routing and PEGASIS.

Figure 9 shows the comparison of average end-to-end delay between the 4-chain, the 2-chain, the single-chain, PEGASIS, and C-PEGASIS. In this figure, we see that the aggregated delay of single chain based scheme is much higher than the other two schemes as all children nodes of the single chain have to forward their data through the same central path. Large transmission distance causes high propagation delay between the end nodes of the chain and the chain head. Nodal delay decreases with the decrease in the number of nodes. Nodal delay is the time taken by a node in data processing. Multi-chain schemes have low end-to-end delay as, if their parent node dies, the distant nodes forward their data to the nearest nodes of the interconnected chains. It reduces the burden on distant forwarder nodes in the same change. It also minimizes the propagation delay of the network. In PEGASIS, the sharp variations in delay are due to the formation of the chain head at different locations. Aggregated network delay falls due to quick death of distant nodes from the sink. As there is no secondary chain head in PEGASIS so nodal delay also increases at the primary chain head. Initially, due to congestion consideration, the nodal delay in case of C-PEGASIS is greater than 2-Chain and 4-Chain based schemes, thus it leads to more end-to-end delay in C-PEGASIS as compared to the two multi-Chain schemes. However, later on, C-PEGASIS shows less end-to-end delay as compared to the two multi-Chain schemes. Although the per node end-to-end delay in C-PEGASIS is relatively high, but this is due to the fact that a relatively fewer number of nodes are alive in C-PEGASIS (during later simulation course) as compared to the two multi-Chain schemes. Thus, the overall end-to-end delay of C-PEGASIS is then lower than the two multi-Chain schemes.

**Figure 10.**
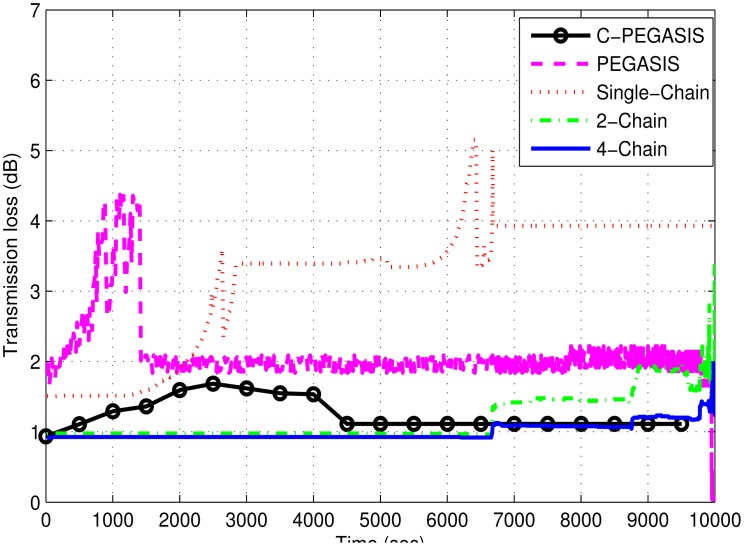
Transmission loss (dB) in 4-chain, 2-chain, single chain-based routing and PEGASIS.

Figure 10 shows the comparison of transmission loss in the discussed schemes, which has been computed by the thorp model. It depends upon the transmission distance, bandwidth efficiency and the attenuation loss of the signal. Larger distance between communicating nodes causes high transmission loss which further increases due to the death of the intermediate nodes. The single-chain based scheme has low transmission loss due to less forwarding distances between the nodes causing high bandwidth efficiency. Moreover, multi-chain schemes have low signal attenuation loss. Transmission loss in the 4-chain based scheme is also less than the 2-chain based scheme due to a fewer number of transmissions. PEGASIS has more transmission loss due to longer transmission distance between chain head and sink than that of compared schemes. On the other hand, C-PEGASIS shows more transmission loss than the two multi-Chain based schemes, and less transmission loss than PEGASIS and Single-Chain based schemes due to moderate communication distance in comparison to the other two groups of schemes.

**Figure 11.**
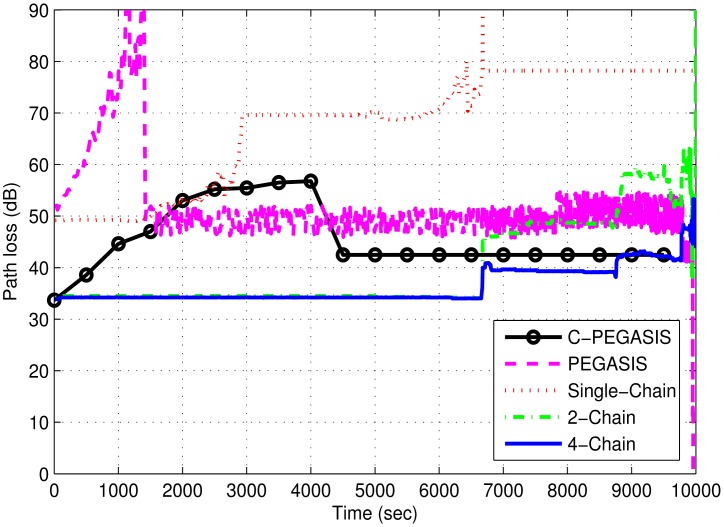
Path loss (dB) in 4-chain, 2-chain, single-chain based routing and PEGASIS.

Path loss of the network depends on a number of factors such as depth of sender and receiver, Euclidean distance and the loss caused by the wave movement. Path loss is computed by using the MMPE model. Figure 11 depicts the comparison of path loss between the chain-based schemes and PEGASIS. It shows similar behavior to transmission loss. In the single-chain scheme, the loss is higher than the other chain-based schemes because of the large transmission distances between the forwarding nodes. However, it decreases with the decrease in number of alive nodes. In the other two chain based schemes, smaller chains reduce the loss of the network, thereby, improving the network performance. However, single-chain has less path loss than PEGASIS due to minimized forwarding distances in the chain. In PEGASIS, path loss keeps on changing due to the random selection of chain heads. Random noise function largely affects the performance of PEGASIS due to the presence of a long link problem. C-PEGASIS shows increase and decrease in path loss till 4000 s and after 4000 s, respectively. The increase in path loss is due to distant communication whereas the decrease is due to the increase in the number of dead nodes. Moreover, the path loss of C-PEGASIS is higher than PEGASIS and Single-Chain schemes, and lower than 2-Chain and 4-Chain schemes, respectively.

**Figure 12.**
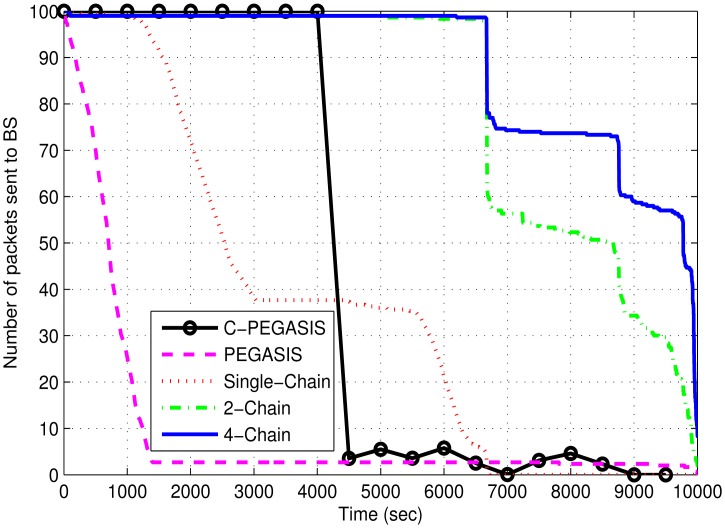
Network throughput in 4-chains, 2-chains, single-chain based routing and PEGASIS.

Figure 12 shows the comparison of network throughput of 4-chain, 2-chain, single-chain based routing schemes and PEGASIS. Network throughput in single-chain based scheme quickly decreases due to the death of forwarding nodes, which have extra load of forwarding data. It also isolates the end nodes of the chain causing the loss of their data packets. In the other two chain based routing schemes, network throughput is much higher due to less burden on the nodes of the chains. The 4-chain based scheme performs better than the 2-chain based scheme due to the balanced load distribution of the intermediate nodes of the chains. Network throughput of PEGASIS and C-PEGASIS quickly falls due to the death of intermediate nodes of the chain.

**Figure 13.**
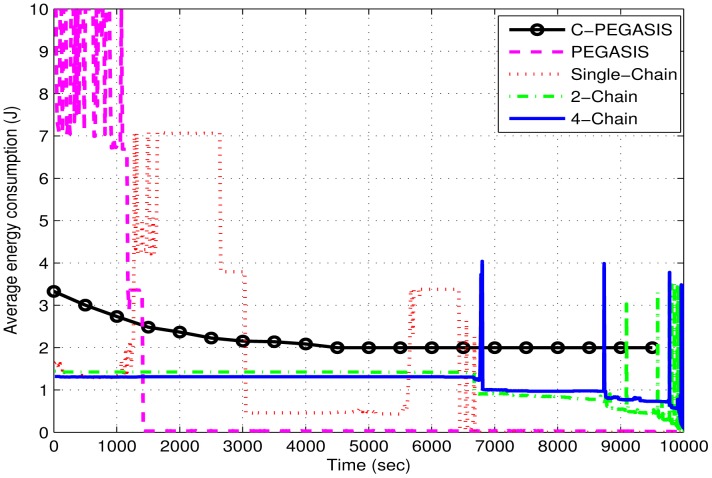
Average energy consumption (J) in 4-chains, 2-chains, single-chain based routing and PEGASIS.

Figure 13 shows the comparison of average energy consumption between 4-chain, 2-chain and single-chain based routing schemes. In the single-chain based scheme, energy consumption is increased suddenly due to death of intermediate nodes in the chain. Death of any intermediate node affects many child nodes and increases their energy consumption. In PEGASIS, average energy consumption is very high at the beginning due to random selection of chain head.

The chain head at a larger distance from the sink consumes a large amount of energy. Reverse transmission in the chain also occurs as the nodes nearer to the sink also forward data towards chain head. The absence of a secondary chain head in PEGASIS causes quick energy depletion of the primary chain head. In C-PEGASIS, the average energy consumption decreases with the passage of time. This decease is due to the death of nodes because energy consumption is directly related with the number of alive nodes.

**Figure 14.**
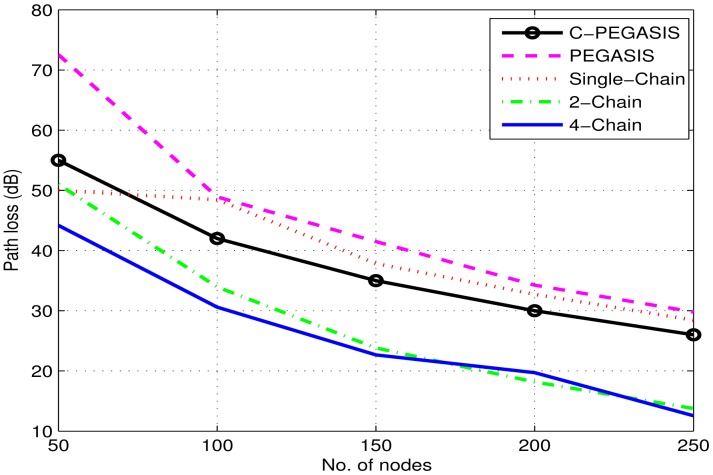
Impact of node density on path loss.

**Figure 15.**
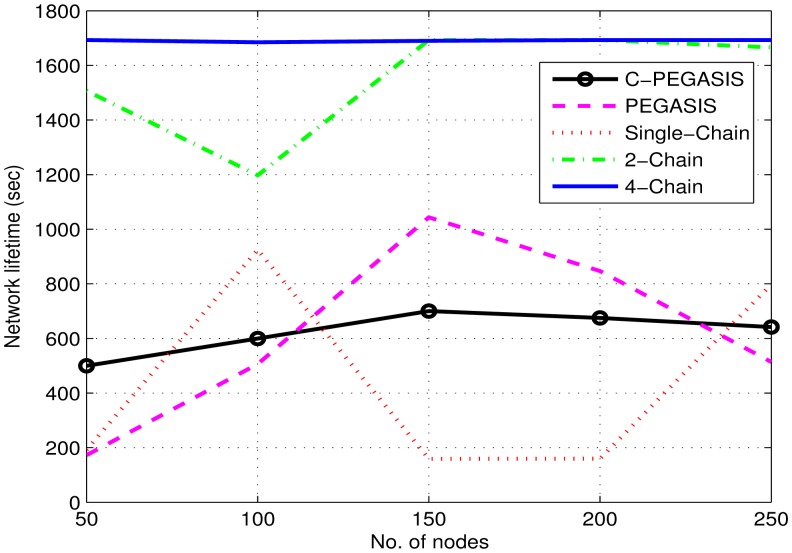
Impact of node density on network lifetime.

**Figure 16.**
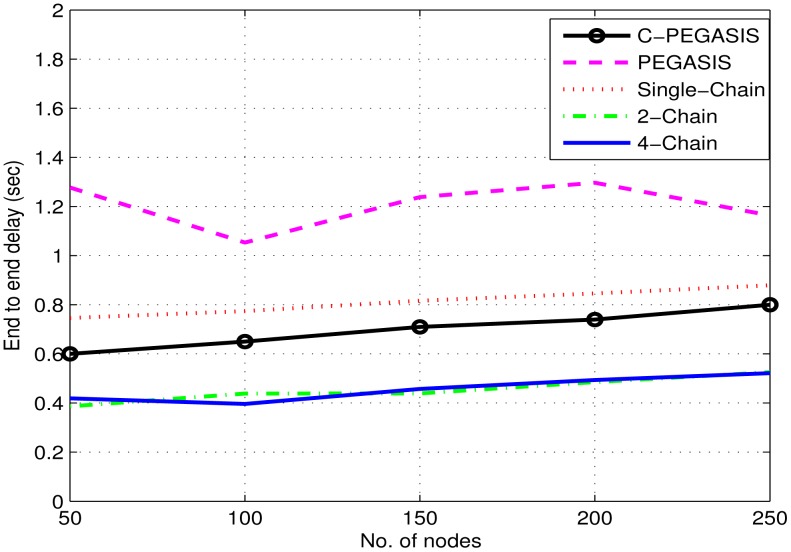
Impact of node density on end to end delay.

Figures 14, 15 and 16 show the impact of node density on path loss, network lifetime, and end-to-end delay. These results are obtained for *E*_0_ = 2*J*. By varying the number of nodes from 50 to 250, all the compared protocols show moderate decay in path loss as shown in Figure 14. This is due to the fact that the communication distance decreases as the number of nodes are increased–leading to moderate decay in path loss. Similarly, a general trend in Figure 15 is first increased and then decreased in the network lifetime as the node density is increased from 50 to 250. The initial increase in network lifetime is due to the fact that increased number of nodes communicate at relatively small distances, which leads to decreased energy consumption and ultimately prolonged network lifetime. However, further increase in node density leads to decreased network lifetime because high node density means increased congestion/interference which leads to high packet drop rate. In such situations, the dropped packets are re-transmitted that require surplus energy, which thus leads to decreased network lifetime. Finally, Figure 16 shows a steady increase in end-to-end delay as the node density is increased. The reason is associated with re-transmissions, *i.e.*, high re-transmission rate means high end-to-end delay.

***Remarks:** In UWSNs, data flows from high depth nodes to low depth nodes and finally towards the sink (at the surface of water). As per our cylindrical network area assumption, addition of another sink at the surface of water would not lead to any significant change in results because the surface area is not too much large. Thus, we suggest a single sink to be placed at the surface of the water. It is also worth mentioning that the routing overhead of our proposed schemes is relatively high as compared to the existing schemes; however, the proposed schemes show significant improvement in terms of networks lifetime as well. Thus, the negative aspect (high routing overhead) is negligible as compared to the positive aspect (prolonged network lifetime)*.

## 8. Conclusions and Future Work

In this paper, we design routing schemes for application-oriented networks. We propose chain-based routing schemes specifically for cylindrical networks. In the 4-chain based routing scheme, there are four interconnected chains to achieve a global optimum solution for data transmission. At first, nodes find the local optimum neighbor and then achieve a global optimum neighbor. In this way, the load is shared between the four chain heads. In a 2-chain based routing scheme, we divide the network into two groups of nodes on the basis of *θ*. Moreover, we also propose the delay computation model for underwater channel communication. We compare the above-discussed schemes with the C-PEGASIS and PEGASIS. The 4-chain based scheme performs better than the other two chain based schemes due to better load balancing and optimal neighbor selection among the sensor nodes.

In future, we aim to devise energy-efficient routing schemes for other application-oriented networks such as sea mine detection, *etc.*, in real experimental test beds.
